# Efficacy analysis of acupuncture and rehabilitation for traumatic spinal cord injury

**DOI:** 10.1097/MD.0000000000041245

**Published:** 2025-01-10

**Authors:** Yue-Bo Jiang, Ling Guan, Ying Li, Man Shu, Bin-Chuan Cui, Zong-Yue Huang

**Affiliations:** a Department of Acupuncture and Moxibustion, The Sixth Medical Center of Chinese PLA General Hospital & Medical School, Beijing, China; b Department of Acupuncture and Moxibustion, The First Medical Center of Chinese PLA General Hospital & Medical School, Beijing, China; c The First Medical Center of Chinese PLA General Hospital & Medical School, Beijing, China.

**Keywords:** acupuncture and rehabilitation, data mining, efficacy analysis, traumatic spinal cord injury

## Abstract

**Background:**

This study investigates the role and efficacy of acupuncture combined with rehabilitation therapy during the recovery phase of patients with traumatic spinal cord injury. Patients hospitalized in the acupuncture department of our center between December 1, 2019, and December 1, 2021, were enrolled.

**Methods:**

Participants were divided into an observation group (acupuncture and rehabilitation therapy) and a control group (rehabilitation therapy alone) based on their treatment sequence. Initially, 15 patients were allocated to each group; however, 3 patients in the observation group and 4 in the control group withdrew, leaving 11 and 12 patients in the respective groups. The observation group received combined acupuncture and rehabilitation therapy, while the control group received conventional rehabilitation therapy. Outcomes were evaluated using the American Spinal Injury Association score and classification, urodynamic data, SF-36 scale, Functional Independence Measure, and Barthel Index, analyzed through data mining techniques.

**Results:**

Posttreatment assessments revealed significant improvements in residual urine volume and detrusor pressure in the observation group (*P* < .05), whereas no significant changes were noted in the control group (*P* > .05). Both groups demonstrated improved motor function after treatment (*P* < .05), with the observation group showing significantly greater improvement (*P* < .05). Quality of life evaluations indicated substantial enhancement in physical pain, energy levels, general health, social functioning, perceived health changes, and mental health in both groups (*P* < .05).

**Conclusion:**

Acupuncture combined with rehabilitation therapy offers significant clinical benefits for patients with traumatic spinal cord injury. This approach effectively alleviates urinary and bowel dysfunction, accelerates motor function recovery, and improves overall quality of life, making it a valuable treatment option worthy of wider adoption.

## 1. Introduction

Approximately 17,000 new cases of spinal cord injury (SCI) occur globally each year, representing a severe traumatic condition characterized by the loss of motor, sensory, and autonomic functions. Due to its limited regenerative ability, SCI remains one of the most challenging conditions to treat. Current treatment modalities include pharmacological intervention, surgical procedures, physical therapy, and rehabilitation. While drug therapy can inhibit local inflammation and oxidation, protect the spinal cord, and prevent neuronal apoptosis, its potential risks and inconsistent clinical outcomes remain significant concerns. Surgical interventions, while sometimes necessary, carry risks of exacerbating injury. Consequently, acupuncture and rehabilitation therapy are increasingly regarded as safer and effective alternatives in clinical practice.

Rehabilitation therapy plays a role in improving motor and sensory recovery in SCI patients but has certain limitations. Acupuncture, a technique targeting specific points on the body, has been shown to facilitate neuronal healing after various central nervous system injuries, including SCI. This underscores its clinical significance.^[1–5]^

Modern medicine recognizes SCI as involving 2 mechanisms: primary injury and secondary injury. Primary injury leads to irreversible nerve damage, while secondary injury, caused by tissue ischemia, hypoxia, and the accumulation of metabolites, results in more extensive damage. Acupuncture and rehabilitation, as traditional Chinese medicine approaches, have undergone continuous refinement through years of clinical practice. Acupuncture is widely used in treating SCI due to its low cost, minimal pain, and simplicity. However, issues such as the standardization of acupoint selection have hindered its effectiveness.

With the rapid development of medical informatics, data analysis has emerged as a valuable tool for guiding clinical practice. This study leverages data mining techniques to analyze clinical literature on acupuncture and rehabilitation for SCI, systematically summarizing treatment methods. By identifying optimal treatment plans for clinical trials, this study aims to offer new insights for future therapeutic strategies.^[6–10]^

Data mining, a process of extracting useful but previously unknown information from large and often disorganized datasets, has applications across industries such as retail, telecommunications, and healthcare. In the medical field, it has proven invaluable for uncovering patterns and generating significant clinical and economic benefits. In this study, Statistical Package for the Social Sciences (SPSS) 18.0 software was employed to analyze clinical data, evaluating the efficacy of acupuncture and rehabilitation for treating traumatic SCI.^[11–16]^

## 2. Background and preliminaries

Limb dysfunction caused by SCI severely impacts patients’ quality of life. Issues such as urinary and fecal incontinence often necessitate continuous family support and frequent hospitalizations due to infections, imposing a significant economic burden. For elderly patients, acupuncture therapy offers unique advantages in managing SCI.

This study utilizes CiteSpace,^[17]^ an advanced visualization tool, to generate integrated knowledge maps from literature on acupuncture and moxibustion for SCI treatment within the China National Knowledge Infrastructure database.^[18]^ This visualization enables researchers to identify the research context, hotspots, and trends in acupuncture-based SCI treatments, guiding future research directions.

A literature search was conducted in China National Knowledge Infrastructure using the keyword “acupuncture AND spinal cord injury” with the “subject” retrieval condition (exact match). The time span ranged from January 1, 1962, to January 1, 2021, yielding 769 articles. After excluding nonrelevant content such as conference notices and newspapers, eligible articles were selected for inclusion.

**Figure 1.**
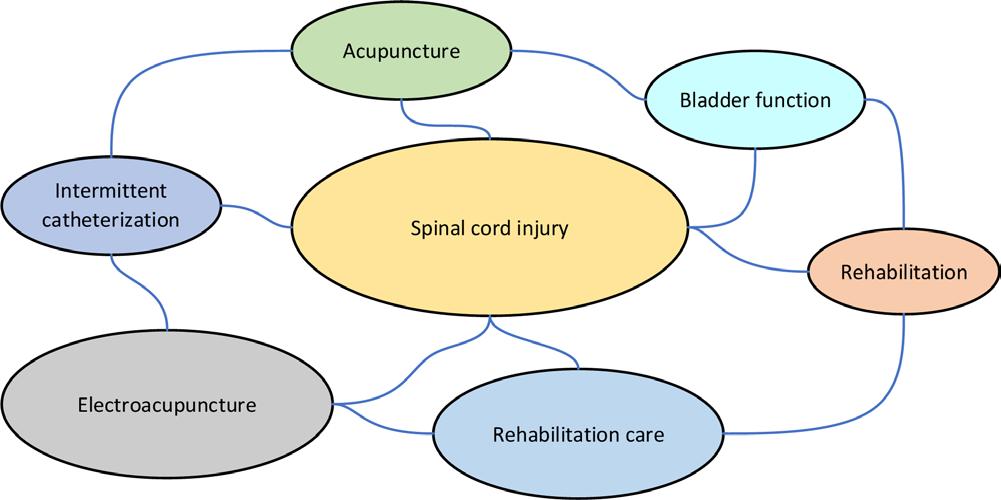
Keyword cluster map illustrating the key research themes in acupuncture and spinal cord injury studies.

**Figure 2.**
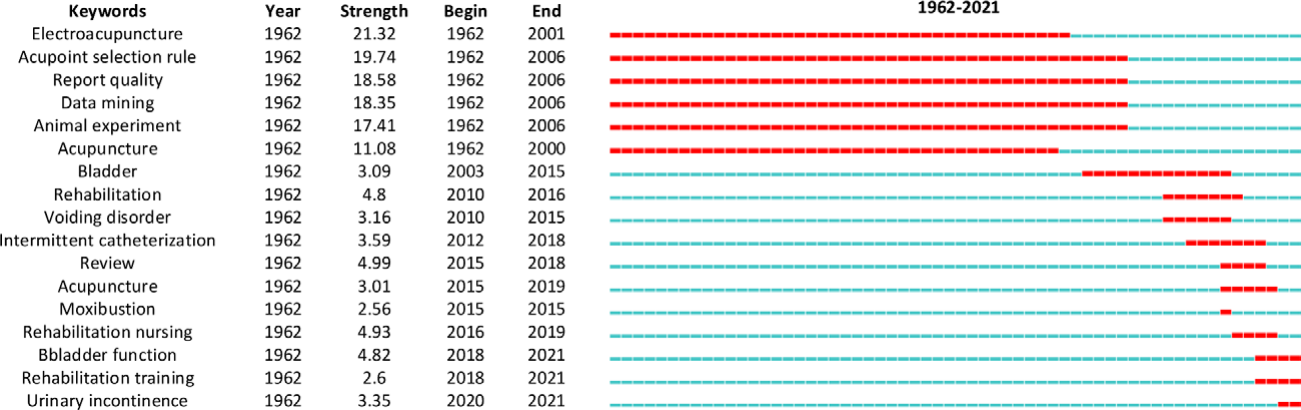
Keyword explosion point map highlighting emerging research topics from 1970 to 2021 in the field of acupuncture for spinal cord injury.

Using keyword clustering, a total of 40 clusters were generated in Figure 1. The top 7 clusters included SCI, electroacupuncture, acupuncture, rehabilitation nursing, bladder function, intermittent catheterization, and rehabilitation training. Explosive keyword trends were identified in Figure 2, including electroacupuncture (1962–2001), acupoint selection rules (1962–2006), and rehabilitation nursing (2016–2019), among others. The most recent trends focused on urinary incontinence (2020–2021).

A timeline analysis revealed that prior to 2006, research predominantly centered on electroacupuncture, acupoint selection, and animal experiments. After 2013, the focus shifted toward integrating acupuncture, moxibustion, and rehabilitation for SCI management, with significant attention given to bladder function, urinary disorders, and urinary incontinence. From 2016 to 2021, advancements in rehabilitation medicine led to increased research interest in rehabilitation nursing and training.

Decades of research in China have validated the efficacy of acupuncture in treating SCI. Based on this foundation, we conducted clinical experiments to further evaluate the benefits of acupuncture for SCI patients.

## 3. Methods

### 3.1. Research subjects

This study included 30 patients with chronic traumatic SCI who were hospitalized in the Central Acupuncture Department of the Chinese People’s Liberation Army General Hospital between December 1, 2019, and December 1, 2021. Patients were randomly assigned to an observation group (acupuncture and rehabilitation therapy) or a control group (rehabilitation therapy alone), with 15 patients in each group initially. Due to the COVID-19 pandemic, 2 patients in the control group withdrew. Additionally, 1 patient in the observation group and 2 in each group withdrew for personal reasons, leaving 12 patients in the control group and 11 in the observation group. All participants provided informed consent prior to enrollment. Baseline characteristics, including age, gender, and duration of disease, were comparable between the 2 groups (Table 1). To ensure reliability, acupuncture therapists, scale evaluators, statisticians, and rehabilitation therapists were assigned specific roles independently.

**Table 1:** Comparison of baseline characteristics of patients between the observation and control groups.

Group	Quantity	Gender		Age	Time	ASIA classification		
		Male	Female			A	B	C
Control group	11	11	2	40.35 ± 12.65	4.42 ± 2.13	6	3	2
Observation group	12	10	2	36.7 ± 13.06	5.38 ± 2.88	5	4	3

ASIA: American Spinal Injury Association: International Standards For Neurological Classification of Spinal Cord Injury.

### 3.2. Inclusion criteria

Participants were included if they met the following criteria: (1) diagnosed with SCI according to American Spinal Injury Association classification grades A to D (revised 2011). (2) Aged 14 years or older with acute or chronic SCI and a disease duration of 3 to 6 months. (3) Preoperative MRI confirmed complete or incomplete SCI. (4) Experienced urinary dysfunction symptoms such as frequent urination, urgency, incomplete urination, dysuria, or urinary incontinence, with residual urine confirmed by B-ultrasound but without evidence of tumors or benign prostatic hyperplasia. (5) Good cardiopulmonary function and tolerance for acupuncture and rehabilitation therapy. (6) Willing and able to participate, complete follow-up, and provide informed consent.

### 3.3. Exclusion criteria

Participants were excluded if they met the inclusion criteria but any of the following conditions: (1) other central or peripheral nervous system disorders. (2) Coexisting diseases with similar symptoms that could confound diagnosis. (3) Severe injuries to other parts of the body. (4) Moderate to severe cognitive impairment. (5) Diagnosed with Alzheimer or Parkinson disease that significantly impairs mobility. (6) Spinal cord injuries caused by tumors or infections. (7) Severe dysfunction of cardiopulmonary, gastrointestinal, hepatic, or renal systems, insulin-dependent diabetes, mental disorders, or pregnancy.

### 3.4. Treatment methods

Acupuncture and rehabilitation group: acupuncture was performed at the following points: Quchi (radial nerve), Neiguan (median nerve), Weizhong (tibial nerve), Yanglingquan (superficial peroneal nerve), Taichong (deep peroneal nerve), Yinlingquan (saphenous nerve), Xuehai (femoral nerve), and Huantiao (sciatic nerve). Two acupoints were alternated daily, and electroacupuncture was applied until a sensation of electric numbness was achieved. Fast needling without retention was used once daily. For patients with neurogenic bladder, acupuncture at the Baliao and Zhongji points was combined with standard rehabilitation therapy. Conventional rehabilitation therapy group: Rehabilitation therapy included: (1) physiotherapy, which strengthened joints, retrained muscles, improved balance and coordination, and facilitated posture changes such as moving from lying to sitting or wheelchair transfers; (2) occupational therapy, which trained patients in daily activities (dressing, eating, mobility) and occupational tasks to help them reintegrate into personal, family, social, and professional life; (3) psychotherapy, which addressed psychological stages such as denial, anger, despair, and adaptation through individual, group, or family therapy; (4) rehabilitation projects, which used braces for standing and walking practice and assistive devices like walkers for functional compensation; (5) clinical rehabilitation, which involved preventive care, symptom management, and therapeutic interventions to facilitate functional recovery; (6) nutritional therapy, which developed tailored diets to meet rehabilitation needs and promote recovery.

### 3.5. Ethical considerations

This study was conducted in compliance with the Declaration of Helsinki for Medical Research and was approved by the Medical Ethics Committee of the First Medical Center of the China Chinese People’s Liberation Army General Hospital (Approval No.: s2020-349-01). Written informed consent was obtained from all participants prior to enrollment. The study was registered in the Chinese Clinical Trial Registry (Registration No.: ChiCTR2100044678).

## 4. Results

The main efficacy evaluation indicators included the American Spinal Injury Association SCI score and classification, SF-36 scale, urodynamic data collection table, Functional Independence Measure, and Barthel index score. Based on statistical requirements and a review of previous literature, the sample size (n = 20) was calculated using a standard formula, and Statistical Package for the Social Sciences (SPSS) software was utilized for data analysis. Data distribution was assessed for normality, and statistical comparisons between groups were conducted using *t* tests or rank-sum tests for quantitative data and chi-square tests or rank-sum tests for qualitative data.

### 4.1. Comparison of urinary function before and after treatment

After treatment, the observation group showed significant improvement in residual urine and detrusor pressure following urination and defecation disorders (*P* < .05), while no significant differences were observed in the control group before and after treatment (*P* > .05). These findings suggest that acupuncture treatment is more effective than rehabilitation therapy alone in improving urinary and fecal functions in patients with spinal cord injuries (Table 2).

**Table 2:** Comparison of urinary function before and after treatment between the observation and control groups.

Group	Quantity	Residual urine		Detrusor pressure		Total efficiency
		Before	After	Before	After	
Control group	11	281.7 ± 77.1*	174.6 ± 91.2	20.9 ± 27.9	42.97 ± 29.9	90
Observation group	12	295.3 ± 80.7**^,^***	273.9 ± 88.9	18.8 ± 25.6	23.78 ± 27.9	46

* *P* < .05, compared with the observation group before treatment.

** *P* < .05 compared to control group.

*** *P* > .05, comparison between the control group and before treatment.

### 4.2. Comparison of motor function before and after treatment

Both groups showed significant improvement in motor function after treatment (*P* < .05); however, the observation group demonstrated a more pronounced improvement compared to the control group (*P* < .05), as shown in Table 3.

**Table 3:** Comparison of motor function improvement before and after treatment in the observation and control groups.

Group	Quantity	FIM score		Barthel score	
		Before	After	Before	After
Control group	11	74.56 ± 30.53	80.11 ± 28.33*	35.56 ± 34.43	41.67 ± 34.55*
Observation group	12	72.32 ± 31.65	81.76 ± 27.32**	32.79 ± 34.98	43.76 ± 34.12**

FIM = Functional Independence Measure.

* *P* < .05, compared with before treatment.

** *P* > .05, compared with control group.

### 4.3. Comparison of mood changes before and after treatment

The SF-36 evaluation of quality of life revealed significant improvements in both groups posttreatment, particularly in physical pain, energy, general health, social functioning, health changes, and mental health (*P* < .05). These results underscore the efficacy of acupuncture in enhancing the overall well-being of patients with spinal cord injuries (Table 4).

**Table 4:** Comparison of mood changes and quality of life improvement (SF-36 evaluation) before and after treatment between the observation and control groups.

Group		Quantity	SP-36	Body pain	Energy	General health	Social function	Health change	Mental health
Control group	Before	11	72.82 ± 9.67	33.8 ± 33.6	34 ± 16.4	29 ± 16.25	36.3 ± 16.3	40.4 ± 18.5	70 ± 29.2
	After		86.4 ± 15.3*	56.9 ± 12.7	47 ± 19.8	47.9 ± 20.6	46.3 ± 21.7	51.6 ± 20.1	20 ± 15.2
Observation group	Before	12	69.95 ± 9.51	33.2 ± 30.5	32.8 ± 17.9	26.7 ± 17.4	35.9 ± 17.2	39.8 ± 19.2	68.2 ± 24.82
	After		74.7 ± 17.96**	55.7 ± 13.1	46 ± 18.8	45.6 ± 19.8	45.8 ± 20.6	49.1 ± 19.7	22.4 ± 16.7

* *P* < .05, compared with before treatment.

** *P* > .05, compared to control group.

## 5. Discussion

The pathophysiology of spinal cord injuries (SCI) can be categorized into primary and secondary injuries. Primary injuries result in irreversible nerve damage, while secondary injuries, including neuroinflammation, axonal displacement, demyelination, parenchymal cavities, and glial scars, exacerbate the damage.^[19,20]^ Currently, there is no definitive cure for SCI due to the limited regenerative capacity of the spinal cord.^[21]^ Treatments often involve a combination of medication, surgery, psychotherapy, physical therapy, and supportive care. High-dose corticosteroids, such as methylprednisolone sodium succinate, can reduce inflammation and oxidative stress, protect the blood-spinal cord barrier, and prevent neuronal death.^[22]^ However, the lack of consensus on standardized corticosteroid usage and concerns over risks and inconsistent outcomes necessitate safer and more effective treatment options for neurological and functional recovery.

Acupuncture therapy, a cornerstone of traditional Chinese medicine, offers a robust theoretical foundation and clinical experience in managing SCI. Acupuncture and moxibustion regulate yin and yang, promote meridian flow, and have shown promising results in alleviating SCI symptoms. Clinically, acupuncture and rehabilitation therapy are particularly effective for motor function recovery. Studies have demonstrated improvements in Activities of Daily Living scores and overall function in patients with traumatic thoracolumbar SCI. Long-term benefits have also been observed with paraplegic 3-needle acupuncture combined with rehabilitation training.^[23]^ Furthermore, acupuncture has been shown to alleviate complications such as pain, neurogenic bladder, pressure ulcers, spasticity, and osteoporosis.^[24–26]^ In this study, body acupoints were used to enhance motor function, potentially activating the neurofeedback mechanism to repair injured areas. Further animal studies are needed to elucidate the underlying repair mechanisms.

### 5.1. The impact of acupuncture combined with rehabilitation on daily motor function

Improvement in daily motor function directly reflects the independence of motor abilities and self-sufficiency in the lives of SCI patients, instilling confidence in their early reintegration into social activities. Acupuncture has demonstrated advantages in alleviating limb pain and enhancing daily motor function in patients. Rehabilitation therapy, which often emphasizes the importance of early intervention, can be combined with acupuncture during the recovery phase of SCI to alleviate symptoms of limb spasticity and improve overall motor function.

### 5.2. Impact on urinary and fecal dysfunction

Urinary and defecation disorders are among the most challenging complications following SCI, significantly affecting patients’ quality of life and economic independence. Spinal cord injuries impair the advanced voiding center, located in the cerebral cortex and pons, and the primary voiding center in the sacral spinal cord (S2–S4). Trauma-induced SCI, often accompanied by pelvic fractures, may compress S2 to S4 sacral nerves, requiring surgical intervention. The Baliao acupoints are located at the sacral foramina, where the autonomic nerves, pelvic floor nerves, and somatic nerves converge, playing a crucial role in regulating the function of the detrusor muscle. The urethral striated muscle, external urethral sphincter, and bladder detrusor muscle are the primary targets innervated by these neurons, coordinating the physiological processes of the urinary system. This study highlighted the efficacy of Baliao point in improving urination and defecation functions. Acupuncture effectively enhanced urinary flow rate, residual urine volume, detrusor strength, and bladder compliance, enabling some patients to abandon urinary bags and reduce intermittent catheterization. These improvements directly enhanced patients’ quality of life.

### 5.3. Psychological benefits of acupuncture and rehabilitation therapy

Psychological well-being is a significant concern for SCI patients, particularly those with a prolonged disease course. Negative emotions stemming from altered social roles, physical disabilities, and financial strain often hinder recovery.^[27]^ Acupuncture and rehabilitation therapy facilitate face-to-face interactions, fostering patient–doctor communication and enhancing patients’ confidence in recovery. This integrative approach improved mental health, motivated active participation in treatment, and expedited recovery. A questionnaire survey revealed that patients undergoing acupuncture and rehabilitation therapy exhibited greater confidence in treatment and societal reintegration, further emphasizing the psychological benefits of these interventions.

## Author contributions

**Conceptualization:** Yue-Bo Jiang.

**Data curation:** Yue-Bo Jiang, Ling Guan, Ying Li, Man Shu, Bin-Chuan Cui, Zong-Yue Huang.

**Formal analysis:** Yue-Bo Jiang.

**Funding acquisition:** Yue-Bo Jiang.

**Investigation:** Yue-Bo Jiang.

**Methodology:** Yue-Bo Jiang.

**Project administration:** Yue-Bo Jiang.

**Resources:** Yue-Bo Jiang.

**Software:** Yue-Bo Jiang.

**Supervision:** Yue-Bo Jiang.

**Validation:** Yue-Bo Jiang.

**Visualization:** Yue-Bo Jiang.

**Writing – original draft:** Yue-Bo Jiang.

**Writing – review & editing:** Yue-Bo Jiang.
